# Sustained reduction in prevalence of lymphatic filariasis infection in spite of missed rounds of mass drug administration in an area under mosquito nets for malaria control

**DOI:** 10.1186/1756-3305-4-90

**Published:** 2011-05-25

**Authors:** Sammy M Njenga, Charles S Mwandawiro, C Njeri Wamae, Dunstan A Mukoko, Anisa A Omar, Masaaki Shimada, Moses J Bockarie, David H Molyneux

**Affiliations:** 1 Kenya Medical Research Institute (KEMRI), Mbagathi Road, Nairobi, Kenya; 2 Kenya Methodist University, Meru, Kenya; 3 Ministry of Public Health and Sanitation, Nairobi, Kenya; 4 Nagasaki University Institute of Tropical Medicine, Nagasaki University, Nagasaki, Japan; 5 Centre for Neglected Tropical Diseases, Liverpool School of Tropical Medicine, Liverpool, UK

## Abstract

**Background:**

The Global Programme to Eliminate Lymphatic Filariasis (GPELF) was established by the World Health Organisation (WHO) in 2000 with the goal of eliminating lymphatic filariasis (LF) as a public health problem globally by 2020. Mass drug administration (MDA) of antifilarial drugs is the principal strategy recommended for global elimination. Kenya launched a National Programme for Elimination of Lymphatic Filariasis (NPELF) in Coast Region in 2002. During the same year a longitudinal research project to monitor trends of LF infection during MDA started in a highly endemic area in Malindi District. High coverage of insecticide treated nets (ITNs) in the coastal region has been associated with dramatic decline in hospital admissions due to malaria; high usage of ITNs is also expected to have an impact on LF infection, also transmitted by mosquitoes.

**Results:**

Four rounds of MDA with diethylcarbamazine citrate (DEC) and albendazole were given to 8 study villages over an 8-year period. Although annual MDA was not administered for several years the overall prevalence of microfilariae declined significantly from 20.9% in 2002 to 0.9% in 2009. Similarly, the prevalence of filarial antigenaemia declined from 34.6% in 2002 to 10.8% in 2009. All the examined children born since the start of the programme were negative for filarial antigen in 2009.

**Conclusions:**

Despite the fact that the study villages missed MDA in some of the years, significant reductions in infection prevalence and intensity were observed at each survey. More importantly, there were no rebounds in infection prevalence between treatment rounds. However, because of confounding variables such as insecticide-treated bed nets (ITNs), it is difficult to attribute the reduction to MDA alone as ITNs can lead to a significant reduction in exposure to filariasis vectors. The results indicate that national LF elimination programmes should be encouraged to continue provision of MDA albeit constraints that may lead to missing of MDA in some years.

## Background

The World Health Assembly (WHA) Resolution 50.29 made in 1997 called for elimination of lymphatic filariasis (LF) as a public health problem [1]. Following this resolution, the World Health Organization (WHO) initiated the Global Programme to Eliminate LF (GPELF) and rapid progress has been made since its launching in 2000 [2-4]. The GPELF recommends that consecutive annual rounds of mass drug administration (MDA) be given to all eligible persons until interruption of transmission is achieved. The recommended antifilarial treatment is a combination of albendazole with either diethylcarbamazine (DEC) or ivermectin (Mectizan). Success of MDA depends on interruption of parasite transmission by reducing the prevalence of microfilariae circulating in blood of individuals living in endemic areas [5].

In Kenya, LF is due to *Wuchereria bancrofti *and is endemic in the coastal areas along the Indian Ocean from Lamu District in the north to Msambweni District in the south bordering northern Tanzania. Entomological investigations in the Kenyan coastal region have reported that *Anopheles gambiae sensu lato, An. funestus *and *Culex quinquefasciatus *are the vectors involved in transmission of LF [6,7]. On the east coast of Africa, the urban mosquito, *Culex quinquefasciatus*, is an important vector in cities and large urban settlements. This mosquito is known to breed in a variety of stagnant water habitats where the water has been sufficiently polluted like in cess pits and canals near houses with long standing bathroom sewage water. As you move inland, *Anopheles *mosquitoes become predominant and are the principal vectors in rural coastal villages on the east coast of Africa [8]. Since malaria is also endemic in these setting there are intense efforts to reduce transmission of the infection mainly through increased distribution of insecticide-treated bed nets (ITNs). In Kenya, nets are supplied to malaria endemic communities through different sources including commercial outlets, free distribution by local NGOs, and national health programmes such as sale of highly subsidized ITNs to pregnant women and children under five through government health facilities.

Kenya initiated a National Programme for Elimination of LF (NPELF) in 2002 which was launched in the larger Kilifi District in 2002 and scaled up to include Malindi and Kwale Districts in 2003. It is becoming more and more apparent that national programmes to eliminate lymphatic filariasis in sub-Saharan Africa may be faced with challenges such as decreased funding and social instability which may affect delivery of consecutive rounds of MDA. The Kenyan LF elimination programme, for example, has been unable to provide consecutive annual rounds of MDA in the three districts and scale up implementation to include the other endemic districts mainly due to budgetary constraints. It is therefore necessary to monitor and evaluate the impact of such inadvertently missed MDA rounds to provide information that may be used to make evidence-based programmatic decisions about such programmes.

The area along River Sabaki in Malindi District was previously reported to be a major focus of bancroftian filariasis [9]. In 2001, eight villages in this area were selected for a pilot demonstration research project to monitor the impact of MDA. Treatment followed WHO guidelines using single-dose annual mass treatment with DEC (6 mg/kg) plus albendazole (400 mg) [10]. Baseline studies conducted in January/February 2002 in the area collected data on microfilaraemia, filarial antigenaemia, antifilarial antibodies and LF related disease manifestations [11,12]. Since implementation of the project, we have been monitoring the impact of MDA [13]. This study documented the impact of two consecutive annual rounds of MDA followed by two other rounds not given in consecutive years on LF infection (microfilaraemia and antigenaemia).

## Methods

### Study area

This study was conducted in LF endemic villages situated along River Sabaki in Malindi District of Coast Region, Kenya. The villages are typically rural and situated between 40 and 60 kilometres west of the Indian Ocean in the Nyika plateau. The Nyika plateau is characterized by hot and dry climate for most of the year with low fertility brown sandy soils, low grassland and thorny bush. The annual rainfall is between 500 mm and 700 mm and the area is sparsely populated. This is in contrast to the coastal plains and ranges near the Indian Ocean which have hot and humid climate. Description of the study area has previously been provided [11].

### Study population and design

In 2001, eight villages located in the *W. bancrofti *endemic area along River Sabaki were purposively selected for this study. According to censuses conducted prior to the first MDA the population of the villages ranged between 600 - 900 persons. This was a longitudinal study designed to follow up approximately 200 individuals registered in each village. In each village, meetings known in Kiswahili as *baraza *were conducted to sensitize the members about the project. Individuals were recruited into the study if 5 years or more, not severely ill and if consent was given. Parasitological surveys to monitor the impact of MDA were conducted periodically to determine changes in prevalence of microfilaraemia and filarial antigenaemia. The most recent post-MDA survey was conducted in April/May 2009 in all eight study villages. The protocol for this study was reviewed and approved by the Scientific Steering and Ethical Review Committees of the Kenya Medical Research Institute (KEMRI). The Scientific Committee reviewed the scientific content whereas the Ethics Committee looked at the ethical issues. Informed consent was supposed to be written on an informed consent form, but many study participants and/or their parents/guardians were illiterate. In such situations the study participant added a thumbprint after giving oral consent and a literate witness, present during the informed consent process, signed on the informed consent form on behalf of the participant to confirm that the participant gave consent.

### Mass drug administration (MDA)

The first round of MDA was administered in the 8 study villages by the research team and Malindi District hospital staff in April 2002. Subsequent rounds of treatment were provided to the entire district by the NPELF which scaled up MDA geographical coverage to include Malindi District as its third implementation unit in 2003. Four rounds of MDA have been administered to the eight villages in April 2002 (MDA1), September 2003 (MDA2), March 2005 (MDA3) and December 2008 (MDA4). We previously reported treatment coverage for 2002 and 2003 MDAs among study participants in four study villages to be 83.4% (range 64.9 - 92.7%) and 80.3% (range 64.9 - 87.0%), respectively [13]. Table 1 summarizes treatment coverage among the study group in 2005 and 2008 MDAs in the 8 study villages. To estimate treatment coverage, verbal interviews were conducted with adult members of the study group during household surveys. The NPELF was unable to conduct MDA campaigns in 2004, 2006 and 2007 due to financial constraints.

**Table 1:** Treatment coverage in 2005 and 2008 MDAs among registered study group


	**2005 (3**^**rd **^**MDA)**		**2008 (4**^**th **^**MDA)**	
	
**Village**	**No. eligible**	**No. treated (%)**	**No. eligible**	**No. treated (%)**

Jilore	156	131 (84.0)	89	61 (68.5)
Marikano	256	234 (91.4)	108	91 (84.3)
Magongoloni	140	130 (92.9)	138	91 (65.9)
Mkondoni	244	230 (94.3)	126	108 (85.9)
Mwangatini	219	209 (95.4)	173	138 (79.8)
Burangi	229	224 (97.8)	160	132 (82.5)
Shakahola	212	200 (94.3)	160	68 (42.5)
Chakama	185	184 (99.5)	142	138 (97.2)
All	1641	1542 (94.0)	1096	827 (75.5)

Treatment coverage was estimated from interviews conducted on adult members residing in study households.

### Blood collection, microfilariae examination and ICT test

Blood samples were collected from the study participants for microfilariae and circulating filarial antigen examinations in February 2002 (baseline), March 2003, July 2004, July 2007 and March 2009. Until the 2007 survey, two 100-μl finger prick blood samples were collected from consenting study participants into heparinized capillary tubes between 2000 h and 2400 h for microfilariae detection and filarial antigen testing. One blood sample was transferred into a tube containing 0.9 ml of 3% acetic acid solution and mixed gently. The acetic acid diluted blood specimens were kept at room temperature until the following day when examination and counting of microfilariae were done using the counting-chamber method [14].

The second finger prick blood was immediately tested for filarial antigen using ICT test cards following manufacturer's instructions. The ICT results were recorded as either negative or positive after 10 minutes, and indeterminate where the test bands were difficult for two readers to classify as either positive or negative. In 2009 survey, venous blood samples were collected during the day and using disposable micropipets, 100-μl blood samples transferred onto the ICT test cards. Only persons who were antigen-positive using the ICT test were also tested for microfilaraemia. Approximately 3 ml of venous blood samples were collected from each study participant into EDTA tubes in 2002 (pre-MDA), 2004 (post MDA2), and 2009 (post MDA4) surveys for preparation of plasma samples to be used for serological studies related to this study.

### Mosquito net survey

Data used for assessing net ownership in the study area come from a survey conducted in 2008 in four of the study villages where mosquitoes were sampled in a study to assess transmission of LF using molecular methods. A structured questionnaire on ownership and use of nets was administered to heads or other adult members of households selected for mosquito sampling.

### Data management and analysis

Data were recorded into data collection forms while in the field and transferred into computer immediately upon return to Nairobi. Statistical analyses were done using SPSS statistical software version 12.0.1 (SPSS Inc., Chicago, IL, USA). The Pearson Chi-square (χ^2^) test was used to compare proportions including prevalence of microfilaraemia and antigenaemia. Microfilarial counts per ml of blood (mf/ml) were log-transformed and the Student t-test used to compare means at baseline and after the four rounds of MDA. The intensities of microfilariae in the study villages were expressed as geometric mean intensity (GMI) in all individuals examined for microfilariae (including those negative for microfilariae). Differences were considered significant when P was < 0.05. The Strengthening the Reporting of Observational Studies in Epidemiology (STROBE) statement was considered when reporting the findings of this study [15].

## Results

### Changes in microfilaraemia

**Figure 1:**
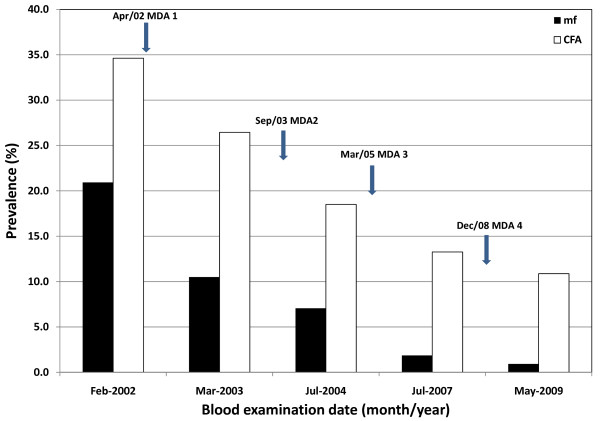
**Trends of prevalence of microfilariae (mf) and circulating filarial antigen (CFA) based on ICT test**. During the 2009 survey, the prevalences of microfilaraemia and antigenaemia were found to have significantly decreased by 95.7% (from 20.9% to 0.9%; P < 0.0001) and 68.8% (from 34.6% to 10.8%; P < 0.0001), respectively.

The overall prevalence of microfilaraemia in the eight study villages in January 2002, before the implementation of MDA, was 20.9%. Since the introduction of MDA in April 2002 a decrease in the prevalence of microfilaraemia has been recorded in each post-MDA survey. The changes in microfilariae prevalence in each village during the study period are summarized in Figure 1 and Table 2. When compared with the 2002 baseline (pre-MDA) data, the overall reduction in prevalence of microfilariae by 2009 was 95.7%. By 2007, there was no microfilariae positive person identified in Chakama village and by 2009 there were no positive persons detected in this and two other villages namely, Burangi and Magongoloni. Further, of the total 1079 persons examined in 2009, microfilariae were detected in only 10 individuals (0.9%). Additionally, the overall GMI of microfilariae in all individuals examined for microfilariae decreased by 98.5% (91.9 - 100%) from 2.75 mf/ml of blood (2.18 - 3.40 mf/ml) in 2002 to 0.04 mf/ml (0 - 0.18 mf/ml) in 2009 (P < 0.001).

**Table 2:** Prevalence of microfilaraemia before and after 4 rounds of mass drug administration (MDA)


	**No. mf positive/No. examined (% mf positive)**				
		
**Village**	**2002 (Pre-MDA)**	**95% CI (%)**	**2009 (Post 4 MDAs)**	**95% CI (%)**	**% Decrease**

Mwangatini	41/180 (22.8)	16.7 - 28.9	2/171 (1.2)	0.6 - 5.0	94.7
Burangi	44/194 (22.7)	16.8 - 28.6	0/159 (0.0)	0.0 - 2.1	100.0
Shakahola	28/157 (17.8)	11.8 - 23.8	2/156 (1.3)	0.0 - 3.7	92.7
Chakama	32/148 (21.6)	15.0 - 28.2	0/139 (0.0)	-	100.0
Jilore	32/173 (18.5)	12.7 - 24.3	3/85 (3.5)	1.0 - 11.8	81.1
Marikano	44/192 (22.9)	17.0 - 28.8	2/106 (1.9)	0.0 - 5.7	91.7
Magongoloni	44/194 (22.7)	16.8 - 28.6	0/138 (0.0)	0.0 - 2.1	100.0
Mkondoni	32/181 (17.7)	12.1 - 23.3	1/125 (0.8)	0.0 - 4.9	95.5
All	297/1419 (20.9)	18.8 - 23.0	10/1079 (0.9)	1.2 - 2.6	95.7

The ages of study participants in the respective survey years were used to summarize changes in the prevalence of microfilaraemia by age in the study villages. As shown in Table 3, there was a significant decrease in microfilariae prevalence in all age groups. There was no microfilariae positive person detected in the age group below 11 years of age in the 2007 and 2009 surveys. Further, except for one person with microfilaraemia in the 11-20 year age group, all the other microfilaraemic persons were aged more than 30 years in the 2009 survey.

**Table 3:** Prevalence of microfilariae (mf) by age before and after 4 rounds of mass drug administration (MDA)


	**No. mf positive/No. examined (% mf positive)**				
		
**Age group (years)**	**2002 (Pre-MDA)**	**95% CI**	**2009 (Post 4 MDAs)**	**95% CI**	**% Decrease**

< 11	30/350 (8.6)	5.6 - 11.5	0/220 (0.0)	-	100.0
11 - 20	76/415 (18.3)	14.6 - 22.0	1/402 (0.2)	0.0 - 1.0	98.9
21 - 30	55/228 (24.1)	18.5 - 29.7	0/108 (0.0)	-	100.0
31 - 40	43/150 (28.7)	21.5 - 35.9	3/127 (2.4)	0.0 - 5.1	91.6
41 - 50	40/136 (29.4)	21.7 - 37.1	3/106 (2.8)	0.0 - 5.9	90.5
> 50	53/140 (37.9)	29.9 - 45.9	3/116 (2.6)	0.0 - 5.5	93.1
All	297/1419 (20.9)	18.8 - 23.0	10/1079 (0.9)	0.3 - 1.5	95.7

### Changes filarial antigenaemia

Figure 1 summarizes the overall change in filarial antigen prevalence based on ICT test in the study area. In general, decrease in prevalence of filarial antigenaemia was recorded during each follow up survey and roughly paralleled the decrease in microfilaraemia. The prevalence of filarial antigenaemia declined from 34.6% in February 2002 to 10.8% in May 2009, which represented a 68.8% (range 57.2% - 77.5% in the individual villages) reduction (P < 0.01). Details of changes in prevalence of filarial antigenaemia in the eight study villages are summarized in Table 4. Of 1096 persons examined for filarial antigenaemia in the 2009 survey, however, there were 38 individuals with indeterminate results. The test bands for these indeterminate cases appeared too faint and it was difficult to record the results as either positive or negative even after giving the cards to a second reader (that is, there was no consensus). The changes in prevalence of filarial antigenaemia by age are shown in Table 5. Unlike microfilaraemia, antigenaemia was detected in children aged less than 11 years in all the years. The greatest decline in prevalence of antigenaemia was seen in individuals aged below 21 years old.

**Table 4:** Prevalence of circulating filarial antigenaemia (CFA) before and after 4 rounds of mass drug administration (MDA)


	**No. CFA positive/No. examined (% CFA positive)**				
		
**Village**	**2002 (Pre-MDA)**	**95% CI (%)**	**2009 (Post 4 MDAs)**	**95% CI (%)**	**% Decrease**

Mwangatini	71/195 (36.4)	29.6 - 43.2	15/164 (9.1)	4.7 - 13.5	75.0
Burangi	69/199 (34.7)	28.1 - 41.3	12/153 (7.8)	3.6 - 12.0	77.5
Shakahola	51/157 (32.5)	25.2 - 39.8	13/156 (8.3)	4.0 - 12.6	74.5
Chakama	44/148 (29.7)	22.3 - 37.1	16/134 (11.9)	6.4 - 17.4	59.9
Jilore	66/172 (38.4)	31.1 - 45.7	13/83 (15.7)	7.9 - 23.5	59.1
Marikano	65/196 (33.2)	26.6 - 39.8	15/106 (14.2)	7.6 - 20.8	57.2
Magongoloni	85/198 (42.9)	36.0 - 49.8	20/138 (14.5)	8.6 - 20.4	66.2
Mkondoni	50/182 (27.5)	21.0 - 34.0	11/124 (8.9)	3.9 - 13.9	67.6
All	501/1447 (34.6)	32.1 - 37.1	115/1058 (10.8)*	8.9 - 12.7	68.8

*excluding 38 individuals who had indeterminate results in 2009

**Table 5:** Prevalence of circulating filarial antigenaemia (CFA) by age group before and after mass drug administration (MDA)


	**No. CFA positive/No. examined (% CFA positive)**				
		
**Age group (years)**	**2002 (Pre-MDA)**	**95% CI (%)**	**2009 (Post MDA4)**	**95% CI (%)**	**% Decrease**

< 11	72/364 (19.8)	15.7 - 23.9	3/217 (1.4)	0.0 - 3.0	92.9
11 - 20	131/423 (31.0)	26.6 - 35.4	20/396 (5.1)	2.9 - 7.3	83.5
21 - 30	82/229 (35.8)	29.6 - 42.0	19/106 (17.9)	10.6 - 25.2	50.0
31 - 40	72/151 (47.7)	39.7 - 55.7	24/125 (19.2)	12.3 - 26.1	59.7
41 - 50	63/138 (45.7)	37.4 - 54.0	20/105 (19.0)	11.5 - 26.5	58.4
> 50	81/142 (57.0)	48.9 - 65.1	29/109 (26.6)	18.3 - 34.9	53.3
All	501/1447 (34.6)	32.1 - 37.1	115/1058 (10.8)*	8.9 - 12.7	68.8

*excluding 38 individuals who had indeterminate results in 2009

### Effects of MDA on children born after implementation of MDA

Children born since the implementation of MDA were aged less than 8 years during the 2009 survey. This group was compared with children of the same age group at each parasitological survey as shown in Table 6. Before MDA in 2002, prevalence of microfilariae in children below 8 years was 4.6%. There was no microfilariae positive child below 8 years of age after two consecutive rounds of single-dose annual MDA given in 2002 and 2003. The prevalence of circulating filarial antigen had also declined to 0% among children aged below 8 years in 2009, but the decrease was not as fast as that for microfilaraemia.

**Table 6:** Prevalence of microfilaraemia and antigenaemia in children aged below 8 years at each survey


	**No. positive/No. examined (%)**			
	
**Month/year of survey**	**mf**	**95% CI (%)**	**CFA**	**95% CI (%)**

February 2002	7/151 (4.6)	1.3 - 7.9	25/165 (15.2)	9.7 - 20.7
March 2003	1/85 (1.1)	0.0 - 3.3	4/85 (4.7)	0.2 - 9.2
July 2004	0/63 (0.0)	-	1/64 (1.6)	0.0 - 4.7
July 2007	0/119 (0.0)	-	2/120 (1.7)	0.0 - 4.0
May 2009	0/77 (0.0)	-	0/76 (0.0)	-

mf = microfilariae; CFA = circulating filarial antigen by ICT test

### Mosquito net use

Around 70% of the people interviewed in the net survey households conducted in four villages in 2008 reported using a net the night prior to the study (Table 7). Additionally, more than 80% of the persons interviewed reported using a mosquito net every night.

**Table 7:** Mosquito net use in four study villages in a survey conducted in 2008


**Characteristic**	**Village**				
	
	**Jilore**	**Marikano**	**Magongoloni**	**Mkondoni**	**All**

No. of houses	36	39	40	40	155
**Net use on night prior to survey**					
Yes (%)	26 (72.2)	21 (53.8)	33 (82.5)	29 (72.5)	109 (70.3)
No (%)	10 (27.8)	18 (46.2)	7 (17.5)	11 (27.5)	46 (29.7)
**Frequency of net use per week**					
Every night	27 (75.0)	28 (71.8)	39 (97.5)	32 (80.0)	126 (81.3)
*Misses a few nights	6 (16.7)	8 (20.5)	1 (2.5)	8 (20.0)	23 (14.8)
∞Misses most nights	3 (8.3)	3 (7.7)	0	0	6 (3.9)

*Does not use net 1-2 nights per week

∞Does not use net 3 or more nights per week

## Discussion

The prevalence of both microfilaraemia and antigenaemia declined considerably in all the eight study villages after the four rounds of MDA given during the 8-year period of monitoring between February 2002 and May 2009. More pronounced decreases in the prevalence of microfilariae and circulating filarial antigen were observed in children less than 11 years of age compared to the older age groups, and by 2007 there was no microfilaraemic individual detected in this age group. A recent study in Papua New Guinea showed that antibody rate also fell more rapidly after MDA in children <11 years of age than in the total study population [16]. In the current study, with the exception of one person with microfilaraemia in the 11-20 year age group, all the other microfilaraemic persons were aged more than 30 years by 2009. Thus, the results of our study indicate that microfilariae in the villages are currently confined to older persons (aged above 30 years). This observation suggests that the MDA given to the villages despite missing several annual rounds had significant effects on prevalence of LF and protected younger persons from the infection. Further, the finding that the decrease in microfilaraemia was greatest for children compared to adults confirm the suitability of samples collected from children for endpoint programme evaluations.

No microfilaraemic positive individuals were detected in three villages (Chakama, Burangi, and Magongoloni) in the 2009 survey but a significant microfilarial rate was recorded in one village (Jilore, 3.5%). There is need to conduct further investigations in such villages with significant residual microfilarial rates to identify underlying factors that might be hindering the decline in LF infection. Such villages may have unique socio-cultural and behavioural characteristics which may prevent some members of the village to comply with MDA or have a significant proportion of immigrants. Simulation models suggest that the impact of mass treatment depends strongly on several factors including compliance [17]. A recent study conducted in Leogane, Haiti to identify factors responsible for continuing LF transmission after 7 rounds of MDA showed systematic noncompliance to be statistically associated with infection status [18]. The Haiti study concluded that high rates of noncompliance maintain a reservoir of infection which drives LF transmission. Our current method of assessing treatment coverage has limitations because only study participants are asked whether they took antifilarial drugs during the last MDA. It is possible that this group is cooperative because of social mobilization efforts designed to enhance compliance with night blood sampling for microfilarial detection and antigen testing. Such intense social mobilization may also have improved compliance to treatment with resultant greater reduction in LF infection. A meeting conducted to understand why some national programs have been more successful than others highlighted the necessity to develop 'compliance profiles' of villages to identify those groups of individuals who remain 'systematically non-compliant' during MDAs and then to determine the causes of this non-compliance and effective approaches to overcoming it [19]. Where operational research may indicate problems with village compliance, alternative social mobilization strategies should also be developed to reach the non-compliers to ensure success of the LF elimination programme.

Although the overall prevalence of microfilariae had decreased dramatically to 0.9% by 2009, the prevalence of circulating filarial antigen, considered a marker of LF infection and adult worm burden [20,21], was 10.8%. It would be important to understand how adult worms exposed to DEC/albendazole treatment are affected in terms of reproductive potential. Thus although filarial antigen positive individuals (without microfilariae) are currently less important in terms of parasite transmission, it would be useful to continue monitoring changes in this marker of LF infection. A total of 38 indeterminate ICT results were also recorded during the 2009 survey which argue for the need to ensure the quality of the diagnostic test(s) as the programme progresses towards the endpoint phase. An ELISA assay based on antifilarial IgG4 antibodies to the recombinant protein Bm14, like the antigen-based tests, was indicated to be highly specific for lymphatic filariasis and has been proposed for long-term monitoring [22,23]. The Bm14 antibody test has been proposed to be an indicator of filarial infection status and/or exposure to mosquito-borne infective larvae [16] and thus may be useful in endpoint evaluation. Similarly, molecular xenomonitoring (MX) has been proposed as an important diagnostic tool especially for monitoring transmission during LF elimination programmes [24-26]. The combination of approaches as described in the evaluation of the national programme in Egypt [27] provides a template for such studies but may be constrained by both human and financial constraints.

The decline in LF infection reported in our study is dramatic considering that annual MDA was not sustained as recommended by GPELF. The results of our study are in contrast to those reported from a study in Haiti where one missed round of MDA caused a considerable rebound in microfilaraemia [28]. Two rounds of MDA plus albendazole can reduce the intensity of microfilariae in a highly endemic community by over 80% [16]. In areas where there is supplementary vector control, the low levels of microfilarial intensities achieved after one or two rounds of MDA can be maintained even in the absence of further MDA especially if *Anopheles *mosquitoes are the principal vectors [29]. The results of mosquito net survey in our study area indicate relatively high usage of nets in the study villages. No infective mosquitoes were found in an LF endemic area of Papua New Guinea one year after 80% of the community started sleeping under long-lasting insecticide treated nets, despite the fact that MDA had been suspended in the area for over 5 years [30]. The apparent inefficiency in the transmission of LF in our study area may be partly due to the different vector-parasite relationships observed in our study area where *Anopheles *mosquitoes are the main vectors, compared to Haiti where transmission is solely by *Culex *mosquitoes [31]. The phenomenon of facilitation exhibited by *Anopheles *vectors is characterized by a dramatic reduction in transmission intensity following reduction in microfilariae densities. In the case of *Culex *vectors, which exhibit limitation, transmission persists at low microfilariae densities [29]. Our results suggest that national LF elimination programmes should be encouraged to continue provision of MDA albeit constraints that may lead to postponement or even skipping of MDA during some years. The sustained reduction of microfilaraemia and antigenaemia in spite of missed MDA rounds may be due a reduction in microfilariae levels or vector densities below the threshold required for transmission by *Anopheles *mosquitoes.

There have been intense efforts to reduce the burden of malaria in Kenya through the use of effective control tools, mainly ITNs and artemisinin-based combination therapy (ACT). An increase in use of ITNs since 2002, when our study was initiated, has been reported [32]. These malaria-specific control efforts most notable being increased use of ITNs have been associated with a decline in malaria in the area [33,34]. It is expected that the massive distribution of ITNs may provide ancillary health benefits beyond those directly associated to reduction in malaria transmission. Some of these additional benefits are likely to include the concomitant control of other mosquito-borne diseases such as LF. Previous reports from the Solomon Islands, where LF is transmitted by *Anopheles *mosquitoes, showed that malaria control efforts by spraying dichloro-diphenyl-trichloroethane (DDT) resulted in reduced LF transmission [35]. Also, a previous research study in Kwale District in Coast Region, Kenya demonstrated that ITNs have beneficial effect on transmission of LF [7].

The findings on LF infection in the examined children born after implementation of the MDA suggest that transmission of *W. bancrofti *infection in this area may have been interrupted.

## Conclusions

We conclude that the four rounds of MDA given over an 8-year period had dramatic effect on reduction of the prevalence of microfilariae (and circulating filarial antigen), albeit MDA being missed in some years. However, because of confounding variables such as insecticide-treated bed nets (ITNs), it is difficult to attribute the reduction to MDA alone as ITNs can lead to a significant reduction in exposure to filariasis vectors. We therefore recommend evaluation of the role of vector control in LF elimination activities. Addition of vector control methods such as ITNs could provide the extra push needed to stop transmission of LF [18]. There is need to conduct additional surveillance studies employing other sensitive tools such as molecular xenomonitoring and antibody-based assays to further assess status of LF transmission in this study setting.

## Competing interests

The authors declare that they have no competing interests.

## Authors' contributions

SMN participated in the conception and design of the study, data collection and analyses, and drafted the manuscript. CSM participated in the conception and design of the study and data collection. CNW participated in the conception and design of the study and helped to draft the manuscript. MS, DAM and AAO helped in data collection and analyses. MJB helped to draft the manuscript and to interpret the results. DHM participated in the conception and design of the study and to draft the manuscript. All authors read and approved the final manuscript.
